# Automated Firmware Generation for Compressive Sensing on Heterogeneous Hardware

**DOI:** 10.3390/s22218147

**Published:** 2022-10-24

**Authors:** Rens Baeyens, Joachim Denil, Jan Steckel, Dennis Laurijssen, Walter Daems

**Affiliations:** 1 FTI Cosys-Lab, University of Antwerp, 2020 Antwerp, Belgium; 2 Flanders Make Strategic Research Centre, 3920 Lommel, Belgium

**Keywords:** code generation, heterogeneous hardware, model-based design, signal acquisition, embedded design, system engineering, compressive sensing, measurement, synchronization

## Abstract

In this paper, a model-based firmware generator is presented towards complex sampling schemes. The framework is capable of automatically generating a fixed-rate Shannon-compliant acquisition scheme, as well as a variable-rate compressive sensing acquisition scheme. The generation starts from a model definition, which consists of two main components, namely an acquisition sequence to implement and the platform on which the sequence should be implemented. This model is then combined with the specifications to be transformed into a functional firmware. When generating firmware for compressive sensing (CS) purposes, the defined acquisition sequence is automatically generated to implement a pseudo-random sampling scheme in agreement with the defined undersampling factor. The evaluation of the generated firmware is done by means of an example use-case, including a proposed strategy for synchronization between CS setups. This research attempts to reduce the development complexity for embedded CS to lower the threshold towards effective usage in the field.

## 1. Introduction

The development of an embedded acquisition system requires a highly specific skill set, encompassing hardware design, software development, signal processing, system-level design, etc. These are all skills that require specialized profiles that are not always available within a development setting. Consider a system engineer who knows what to accomplish on a system level. The required skill set for the implementation of a full acquisition setup contains many intricate low-level details, such as being able to program and compile a platform-specific firmware and designing a custom PCB, in addition to system-level design skills, especially for heterogeneous platforms, where currently the full potential can only be unleashed by knowing the underlying architecture and processing mechanisms in detail. Besides the development complexity, there is another hurdle: there is no feasible solution for mid-volume production. Currently, there are two feasible scenarios for the implementation of acquisition systems. The first one is feasible for low-volume solutions, and implies the use of a specific DAQ device that is computer controlled. This is typically too expensive as a solution for a larger scale. The second scenario is usable for high-volume solutions and implies the development of a custom PCB and a highly specialized design effort, such as developing a bare-metal firmware or a dedicated acquisition FPGA design. The latter contains an extensive engineering cost that can only be amortized over high volumes. There is a wide range of use cases in between the two options that is currently unexplored terrain. Since the development cost is a big hurdle, a simplification of the development process can lead to a significant gain and prove its purpose in closing the gap between small and high-volume implementations. Combining this simplified software development process with available heterogeneous system-on-chips that can offer high-speed I/O processing leads to a decreased effort for the hardware design process. This combination can lead to a lower development cost, making it feasible to implement custom acquisition devices for specific use cases with a medium batch size.

In an attempt to develop a future-proof reusable framework, the generic parts in the development process have been identified and abstracted using a model-based approach. With the implementation of minimalistic platform-specific libraries, the framework maintains a good level of portability, platform independency and maintainability. Previous research has demonstrated that a generic cross-platform development of acquisition systems respecting the modeled timing requirements can be achieved [1]. This paper explores whether the framework is generic enough to support a complex sampling scheme, i.e., compressive sensing (CS). In the work of O’Connor, an algorithm for embedded CS is proposed [2]. The algorithm is explained in more detail in Section 4. This is a good example of an acquisition scheme that is arduous and error prone if one was to write this from scratch. Due to the use of pseudo-random numbers for the delay between samples, the programmer could easily lose track of the required timings. In addition, discrepancies in the sampling period are hard to be located using traditional debugging methods. This is hard to combine with the requirement for the code to be “on-the-clock-true”. Therefore, an option to generate a CS sequence has been added to the generator framework. Thanks to the possibility to sample below the Nyquist rate [3], the data rate can be reduced significantly when compared to traditional sampling [4], whilst maintaining an accurate reconstruction. This reduction brings forth several advantages, such as a decrease in network congestion, battery consumption and memory usage [2]. Since this approach lowers the platform requirements, it will also reduce the setup cost for a larger number of nodes.

**Figure 1:**
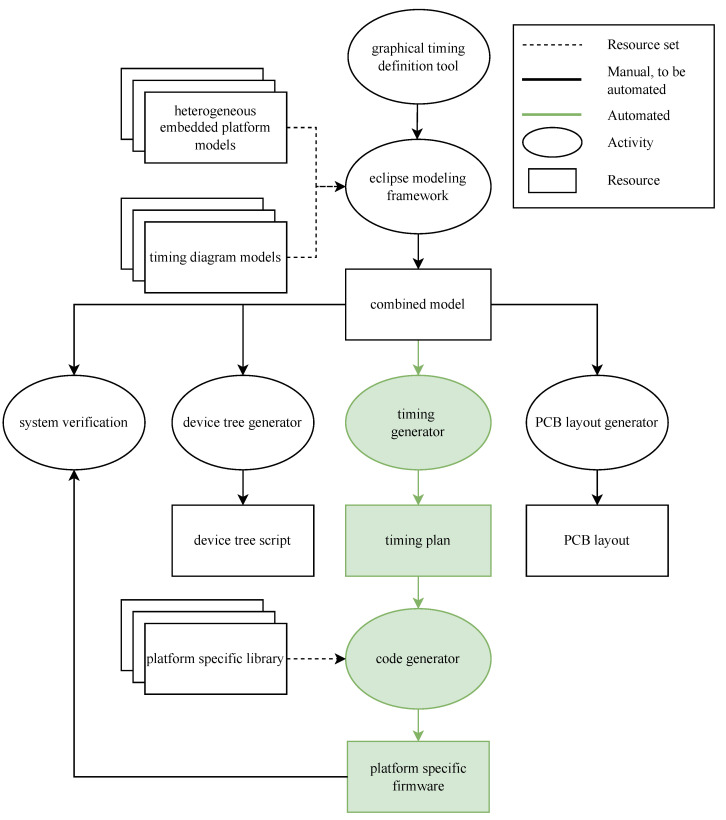
Overview of the proposed framework for model-based code generation for complex sampling schemes. The generation process starts by defining an acquisition sequence using a graphical timing definition tool. This is parsed into the eclipse modeling framework. From here, the acquisition sequence is automatically processed to generate a functional firmware in agreement with the specified acquisition sequence and heterogeneous platform [1].

This paper demonstrates that the proposed framework from our previous work [1] is generic enough to support complex sampling strategies, such as CS, and as such unleashes the power of heterogeneous platforms to the larger public. In Figure 1, an overview of the current status of the framework is presented. For a more detailed view of the framework, the repository containing the model-based code generator and some example models have been made publicly available [5]. This framework attempts to enable system engineers without intricate soft- and hardware knowledge to design and develop acquisition systems. With the model-based code generator simplifying the development of acquisition systems, the generation of complex sampling schemes becomes possible with the only prerequisite being the ability to produce a set of acquisition requirements.

This paper focuses on a complex use case for the model-based code generator to generate data acquisition systems. This paper also demonstrates the genericity for different platforms. As example platforms, an STM32MP157DK2 and a Beaglebone Black were selected, for which elaborated examples can be found in the publicly available repository [5].To maintain the desired genericity whilst expanding the framework, an abstraction was made between the generic parts and the necessary platform-specific definitions. This is achieved with the use of minimal platform-specific libraries for the coprocessors. These libraries are currently developed for Cortex M4 coprocessors, as well as RISC coprocessors such as the two PRU coprocessors implemented on a Beaglebone Black. Since the assumption is made that the main processor of the heterogeneous platform runs some form of an Operating System (OS), the code developed for the main processor is a mainly generic program with minimal platform dependency, in the firmware generated for the coprocessor(s) the generic part is separated from the specific part using the developed platform specific libraries. The generation of these bits of software leads up to a functional acquisition setup fully respecting the modeled acquisition sequence.

In Section 2, an in-depth explanation of the model-based code generator is given. To fully comprehend the next step of generating an acquisition sequence for CS, an introduction to CS is provided in Section 3. Section 4 gives a detailed view into the code generation for embedded CS. As proof of the functionality and correctness of the generator, a use case was elaborated, which is demonstrated in Section 5. Section 6 discusses a technique for synchronization of multiple acquisition setups. Section 7 summarizes some suggestions for future work. Lastly, Section 8 concludes the demonstrated work.

## 2. The Model-Based Code Generator

**Figure 2:**
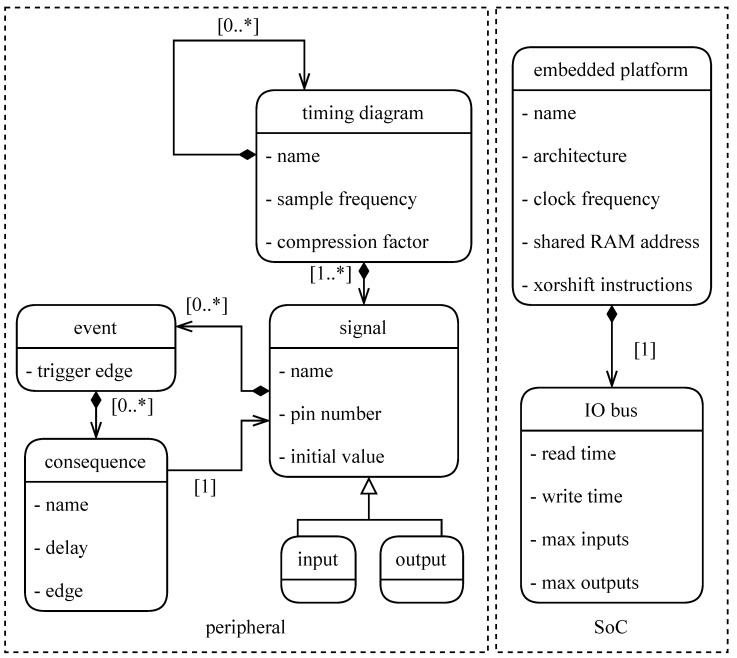
Simplified overview of the combined model representing the acquisition system.

To enable system engineers without software knowledge to generate a functional acquisition setup, the actual coding has to be abstracted from the developer. The ideal way to achieve this, is by using a model-based approach. The reason for this is that a model gives a more intuitive insight and if properly designed, is human-readable without requiring programming language knowledge. To develop the model, the Eclipse Modeling Framework was used [6]. The developed model consists of two main components: firstly, a definition of the sequence of events necessary to accomplish an acquisition and/or actuation (basically a collection of signals and their event triggers). This model definition can be deduced from the timing diagram, which can typically be found in the ADC/DAC datasheet. Secondly, a definition of the platform that will be used for acquisition or actuation along with its specifications such as, e.g., clock frequency, memory size, etc. This combined model, as can be seen in a simplified form in Figure 2, contains all the information that can not be defined in a generic manner. Two independent components are necessary for a functional acquisition system, namely a timing diagram (on the left in Figure 2) and an embedded platform (on the right in Figure 2). The timing diagram consists of a set of signals, which in turn contains a sequence of events that trigger signal transitions. The definition of the event sequence only includes a single acquisition sequence, such that the timing can be matched to the defined sampling frequency. The most important features of the embedded platform are the architecture and the clock frequency. The architecture definition is used to select the correct compiler and combine the correct libraries with the generated timing. The clock frequency is used to match the timing diagram with the embedded platform. Furthermore, the IO timings are used to evaluate whether all defined signal transitions are feasible on the desired platform. From the specifications of these two components a fully functional firmware is generated using model-to-text generator tool Acceleo [7]. This way the model serves as an abstraction layer between the designer, e.g., a system engineer, and the actual required programming knowledge. Thanks to the modular construction, the component models can be interchanged with different platforms and/or acquisition sequences, enabling easy reusability and portability of the defined components. From this step onwards, all steps are performed behind the scenes by the model transformation. This means that the abstraction level lies at the model definition. The complete version of the presented class diagram can be found in the publicly available repository [5].

## 3. Compressive Sensing: A Brief Introduction

### 3.1. Sensing Mechanism

Conventional approaches to sampling signals or images follow Shannon’s theorem stating that the sampling rate must be at least twice the maximum frequency present in the signal to be captured (also referred to as the Nyquist rate) [3]. The sensing mechanism can be described as

(1) $$y_{k}=\langle f,\varphi_{k}\rangle ,\;k=1,2,\ldots ,n.$$

This states that the object *f* is correlated to be acquired with *n* sensing waveforms $\varphi_{k}\left(t\right)$. The sensing waveforms used are typically Dirac delta functions (spikes). Next, $y_{k}$ is organized as columns of Φ such that it can be written in matrix notation:

(2) $$y_{k}=\Phi \cdot f$$

The theory of CS goes against this common wisdom in data acquisition by asserting that in many real applications, a signal or an image can be recovered from *m* samples, with m≪n, as long as the signal content is sparse enough when expressed in a suitable domain [4,8].

### 3.2. Sparsity

Sparsity expresses the idea that the information contained in a signal can be much smaller than it’s bandwidth would suggest, many natural signals have concise representations when expressed in a suitable basis. Assume a vector $f\in R^{n}$ that is expanded in an orthonormal basis (e.g., a Fourier basis) $\Psi =[\psi_{1},\psi_{2},\ldots ,\psi_{n}]$ as follows:

(3) $$f\left(t\right)=\sum_{i=1}^{n}x_{i}\psi_{i}\left(t\right)$$

where *x* is the coefficient sequence of *f* with $x_{i}=\langle f,\psi_{i}\rangle$. If $\psi_{i}$ is organized as columns of Ψ it can be written in matrix notation:

(4) f=Ψ·x

The implication of sparsity is now clear: when a signal has a sparse expansion, multiple $x_{i}$ will be so small that they can be discarded without much loss of accuracy. Formally, consider the $f_{s}\left(t\right)$ obtained by keeping only the terms corresponding to the *S* largest values of *x* in the expansion *f*. This vector is sparse in a strict sense since all but a few of its entries are zero. Since Ψ is an orthonormal basis

(5) $$\|f-f_{s}{\|}_{\ell_{2}}={\left\|x-x_{s}\right\|}_{\ell_{2}},$$

and if *x* is sparse or compressible in the sense that sorted magnitudes of $x_{i}$ decay quickly, then *x* is well approximated by $x_{s}$, and the error $\|f-f_{s}{\|}_{\ell_{2}}$ is small. This is obviously not a new principle, since this is the cornerstone of compression technology. However, the fundamental difference between compressive sensing and typical compression is that there is no need to capture *n* samples but only a subset *m*, and as such the compression is frontloaded.

### 3.3. Undersampling and Sparse Signal Recovery

Combining Equations (2) and (4) delivers a setup for recovery of a vector $x\in R^{n}$. An unknown measurement error term *z* is added to the equation,

(6) y=ΦΨx+z.

The combination of the sensing waveform Φ and the orthonormal basis Ψ leads to an *m* x *n* “sensing matrix” providing information about *x* and will be referred to as *A*, leading to:

(7) y=Ax+z.

To estimate the probability for exact recovery using CS, a key notion has proven to be the restricted isometry property (RIP) [9]. When the sensing matrix *A* is engineered to comply with the RIP, a successful recovery is possible. As such, it guarantees that different sparse vectors *x*, will result in different measurements *y*.

The literature on CS describes a large number of approaches towards solving the recovery problem. A listing of these approaches can be found in the work of Needell and Tropp [10], where it is followed by an explanation of their proposed algorithm CoSaMP. CoSaMP is at heart a greedy pursuit, which implies that it builds up an approximation one step at a time, by making locally optimal choices at each step. At the same time, it incorporates ideas from combinatorial algorithms to guarantee speed and to provide rigorous error bounds. The in-depth explanation of the CoSaMP algorithm will not be provided here, since the data reconstruction serves merely as a tool to prove that the generated acquisition firmware is correct.

## 4. Code Generation for Embedded Compressive Sensing

The purpose of this research does not lie in proving the added value of CS, since there is plenty of literature available on that topic [2,4,8,10,11,12,13,14,15,16]. However, significant steps forward can still be made in the development process of acquisition systems implementing the proposed embedded CS strategies [2]. In current approaches, the implementation of a CS strategy is manually programmed and therefore, as mentioned above, arduous and error prone. For this reason an option for CS is added to our model-based code generator [5]. To avoid that the user of the generator has to acquire an intricate knowledge of the foundations of CS, the generator automatically modifies a traditional acquisition sequence in compliance with Shannon’s theorem. This has the additional advantage that the user can easily switch CS on and off without having to redefine the acquisition sequence model. In the research of O’Connor [2] an algorithm is presented for embedded CS. This research differs from most of the existing research in the sense that it presents a CS algorithm, which makes sense to implement on an embedded device. Whereas most of the research starts from a fully acquired dataset and then experiments with imposing a random cherry-picking scheme on the entire set, implementing this on an embedded device would require plenty of memory, whilst most embedded devices are memory-constrained. The algorithm used in the research of O’Connor starts from the assumption that the random cherry-picking from an existing dataset can be replaced with the introduction of a random but limited amount of skipped samples during acquisition. Obviously, this will have an impact on the overall randomness of the CS dataset, and as such this has an impact on the RIP [9]. The assumption implies that the pseudo-random dataset will also comply with the RIP for most signals and as such enable a successful reconstruction. For the calculation of the pseudo-random amount of skipped samples *d*, the assumption is made that for an undersampling factor of *n*, the dataset is still sufficiently random if *d* is uniformly distributed between 0 and 2n−1 skipped samples, with *n* as the compression factor.

(8) $$d\equiv x\;mod\left(2n\right),\;x\in U\left[0,1,\ldots ,2^{16}-1\right]$$

The model generator is developed in such a way that the amount of clock ticks necessary for the defined timing diagram are counted on generation. From that result, the amount of clock ticks of delay required to match the defined sampling frequency is provided by no operation instructions (NOPs). To do this in a constant and stable manner, the exact amount of instructions needs to be known, constant and stable. This calculation has been extended with the amount of instructions necessary for the calculation of *d*. When implementing this on an embedded device’s coprocessor, where every additional action adds a delay, the main difficulty is that adding a pseudo-random number generation between samples adds a certain amount of code. The algorithm for this generation should be constant in execution time, to prevent having to evaluate the timing after every sample. To this end, the calculation of a pseudo-random value *x* is done using an xorshift algorithm as proposed by Marsaglia [17]. This pseudo-random value *x* is then modulo divided by twice the undersampling factor *n*. Due to the combination of the pseudo-random generation and the modulo division of the pseudo-random number *x*, the calculated delay values are uniformly distributed resulting in an undersampling factor *n*. This leads to an average acquisition rate equal to the traditional sample frequency divided by the undersampling factor *n*. Since the xorshift algorithm requires xor and bitwise shift operations -what’s in a name-, it is easily implemented on different embedded platforms. The only platform-specific part caused by this approach is the amount of clock cycles required for the pseudo-random number calculation. This can vary slightly on different processors due to pipelining, memory caching, etc., but can be assumed constant for a specific processor and is therefore defined in the platform model as part of the platform specification. This also implies that once a platform has been implemented in the generator for CS, it can be reused in combination with new or existing acquisition sequences without having to develop any additional platform-specific resources.

## 5. Generated Firmware in Action

To verify the robustness of both the implemented CS strategy and the generated firmware using the model-based code generator, a setup was developed to simulate a real acquisition scenario. The setup uses a National Instruments DAQ unit to generate a test signal. To ensure that the signal is sufficiently sparse to meet the RIP for CS recovery, a combination of three sine waves was used, at 1 kHz, 21 kHz and at 43 kHz. The simplicity of this signal was chosen as such since the theory behind CS and its recovery capabilities are not the main scope of this paper. The use case is intended to demonstrate the time-wise accuracy of the generated firmwares.

This test signal is coupled to a combination of a custom PCB with buffer amplifiers, sampling filters, an ADS8556 ADC [18], and the direct IO inputs of the Beaglebone Black’s coprocessor [19]. This combination was manually developed by Verreycken et al. [20] for classical Nyquist sampling. The firmware for this project was manually developed in approximately 15 working days by an engineer skilled for the job. The development effort invested in this project illustrates that even for acquisition systems without CS, the manual programming and development of such a system is arduous, error-prone and requires intricate low-level hardware and software knowledge. As a first test, the model-based code generator is used to generate a traditional Shannon-conform acquisition sequence for the ADS8556. This firmware has been thoroughly evaluated in previous work [1] and will serve as the ground truth for the CS firmware to be compared with. The development of the ground truth firmware using the code generator took about three hours, including testing. Thus, the code generator offers an improvement with a factor 30 when compared to manual firmware development.

### 5.1. Time-Wise Accuracy

**Figure 3:**
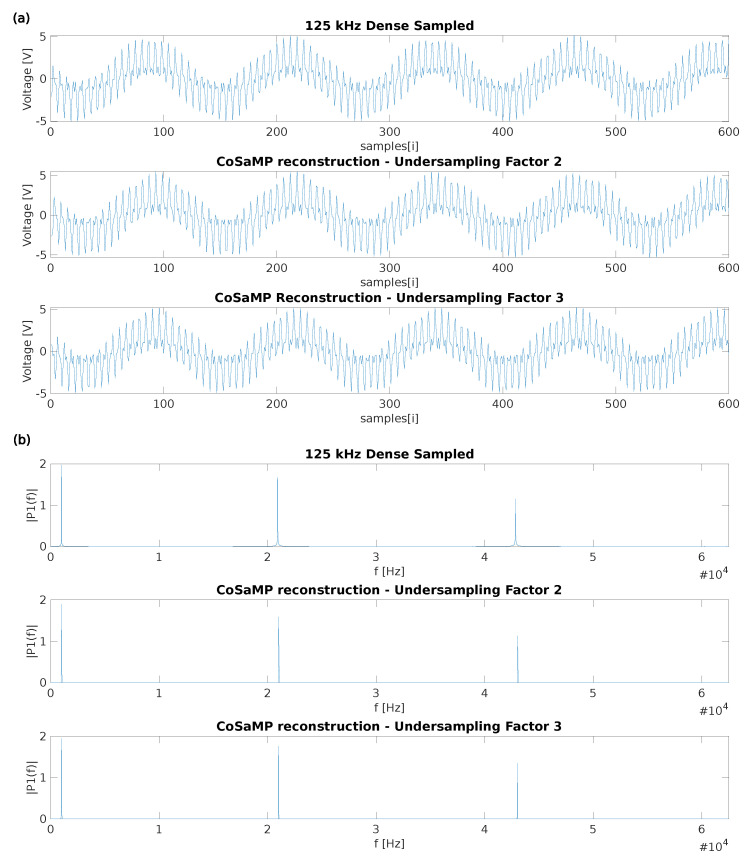
Captures of the test signal using the three auto−generated firmwares. The test signal was a combination of three sine waves with a frequency of 1 kHz, 21 kHz and 43 kHz. To validate the undersampled data, reconstruction was done using the CoSaMP algorithm. In panel (**a**), the time−domain representation can be seen, whereas in panel (**b**), the frequency domain representation is displayed.

To test the CS generation, the CS option was enabled in the generator, which requires practically no additional effort. To prove that the generator functions well for different undersampling factors n whilst maintaining a successful reconstruction, at first two cases were tested, being *n* = 2 and *n* = 3. A comparison of the results using the three auto-generated acquisition firmwares can be seen in Figure 3. As mentioned in Section 3, the undersampled data reconstruction was done using the CoSaMP algorithm [10]. The top figure presents the time domain values for the original signal, as well as its reconstructions for the two tested values of *n*. The bottom figure shows the frequency domain values for the same three configurations. Let us discuss these results. Since the results for all three cases exactly match the generated sine waves’ frequencies (1, 21 and 43 kHz) the time-wise accuracy of the captured samples can be considered “on-the-clock-true”. If a mismatch between the defined and actual sampling frequency would be present, the calculated signal frequencies would be shifted, which is currently not the case. For the lowest frequency (1 kHz) a small difference in peak amplitude is visible for undersampling factor two. To evaluate this effect, different sections of the fully sampled signal were reconstructed. This revealed a certain tolerance on the peak of up to five percent when compared with the dense sampled peak amplitude. This effect is significantly smaller on the higher undersampling factors. This is probably due to the proposed approach not generating a sufficiently random sensing matrix for such a low undersampling factor, and therefore not complying with the RIP. For this reason, a minimum compression factor *n* of three is recommended, to guarantee an accurate reconstruction.

From this comparison, the conclusion can be drawn that the generator is successfully generating firmware for CS purposes. The success of the reconstruction demonstrates that the proposed pseudo-random sampling method is appropriate for CS reconstruction using previously established reconstruction methods [10].

### 5.2. Compression Robustness

**Figure 4:**
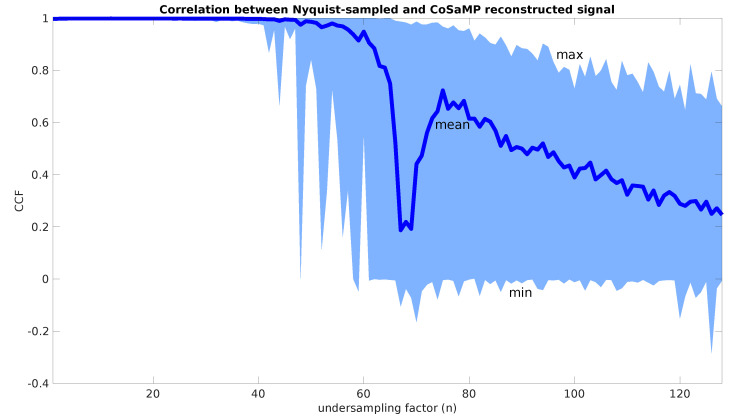
Monte Carlo simulation of the cross-correlation between the Nyquist−sampled test signal and its reconstruction for an undersampling factor *n* ranging from 1 to 128. The bounds for the confidence interval in the figure are the minimum and maximum results from the Monte Carlo simulation, whereas the thick blue line is the mean correlation.

Secondly, to explore the boundaries of CS for the used test signal, the cross-correlation factor between the Nyquist-sampled test signal and its undersampled version is calculated. The undersampling factor for this calculation ranges from 1 to 128. Due to the recovery success being calculated as the likelihood of an accurate reconstruction, a Monte Carlo simulation was used with 100 iterations per undersampling factor, using a different pseudorandom dataset for each simulation [21]. As an indicator for the reconstruction’s success, the minimum, maximum and mean of the cross-correlation factors of each reconstruction with the original Nyquist-sampled signal are used. In Figure 4, the results are demonstrated. The figure demonstrates that up until an undersampling factor of forty-four, the reconstruction achieves a mean value of 99.5% cross-correlation with the original signal. Above an undersampling factor of 44, the mean value of the cross-correlation factors drops below 99.5%. Due to the sparsity constraints inherent to CS, this is of course a signal specific result, and as such cannot be used to draw fixed conclusions on general limitations of the undersampling factor. However, including this simulation with a well-selected range of desired signals to be captured with the generated firmware, can help the designer to make an appropriate choice for the undersampling factor definition in the generator model.

## 6. Synchronization between CS Setups

**Figure 5:**
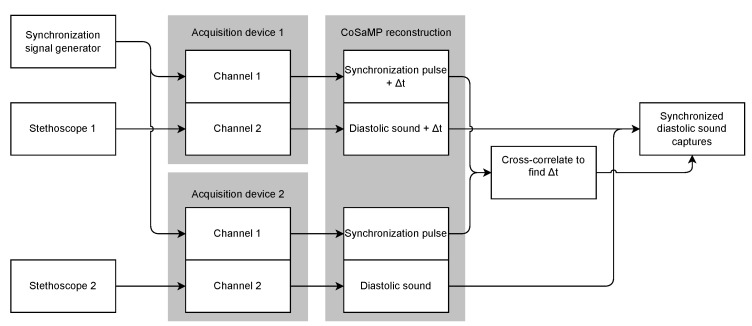
Setup for the demonstration of the proposed synchronization approach using a pseudo-random block pulse generated using a signal generator.

Synchronization is a recurrent issue in measurement setups due to the multimodality of typical sensing setups (e.g., video, audio and motion) [22,23,24,25]. To validate the proposed synchronization approach, a test scenario is elaborated using a combination of two identical acquisition devices, two digital stethoscopes capturing diastolic sound and a synchronization signal. The stethoscopes are each connected to a separate acquisition device. A second channel on the acquisition device is used to capture the synchronization signal as portrayed in Figure 5. The use case is designed to evaluate and address the issue of synchronization for CS in particular.

All channels have been captured with an undersampling factor *n* of 3 and a defined sampling frequency of 125 kHz, that leads to an average sampling frequency of 41.667 kHz when applying Equation (8) found in Section 4. The synchronization signal is a pseudo-random block pulse generated using a National Instruments USB-6363 DAQ. This approach was chosen due to previous success in synchronization research from Laurijssen et al. [26]. This research formulated the minimum and maximum period between signal transitions of the pseudo-random synchronization block pulse. However, this approach assumes traditional Nyquist sampling. Since it is crucial that no signal transitions are missed, the minimum and maximum periods are impacted by the undersampling factor. The minimum period or lower boundary has to be a value in proportion with the maximum number of skipped samples. Combining the conclusion from the research of Laurijssen et al. with the maximum number of skipped samples (two times the undersampling factor), the assumption is made that a proper synchronization can be guaranteed starting from a minimal transition time (in sample periods) of at least four times the undersampling factor. To determine the maximum period or upper boundary, a similar assumption is made. The research from Laurijssen et al. suggested that at least ten transitions need to be present in the synchronization signal. As such the upper boundary is dependent on the captured signal length. Dividing the captured signal length by the product of the minimum amount of transitions with twice the undersampling factor gives us the assumed upper boundary for successful synchronization. These assumptions are discussed and tested in detail in Section 6.1.

**Figure 6:**
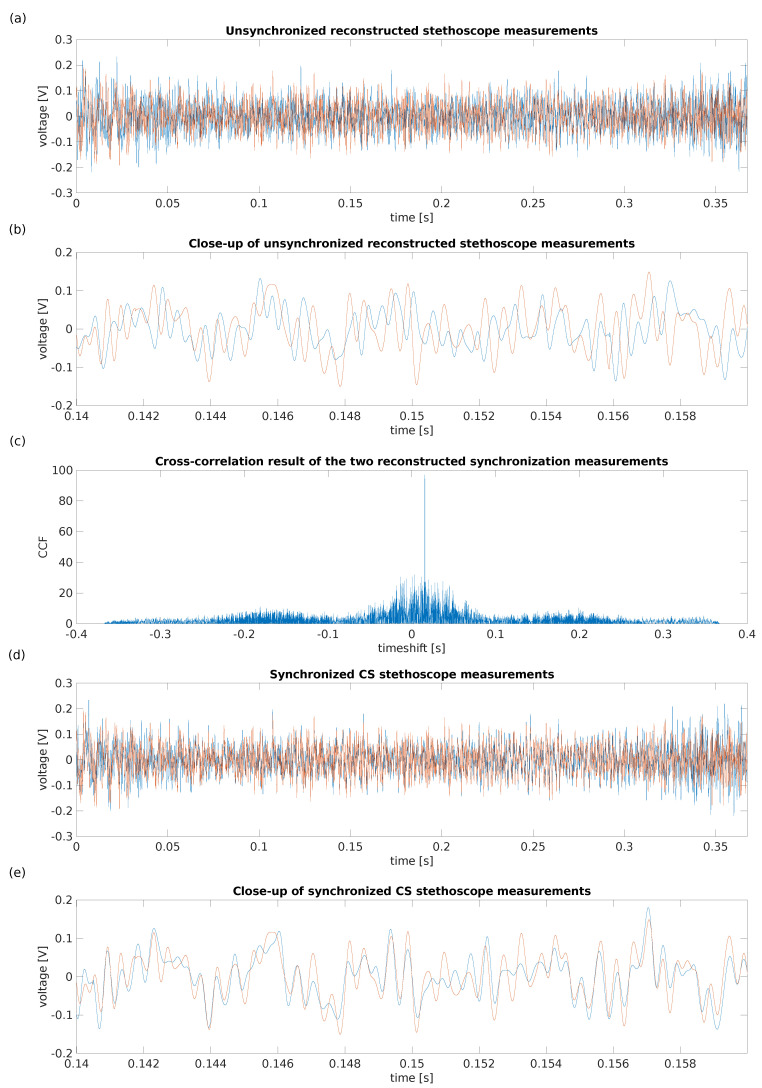
Summary of the results of the CS synchronization setup. Subfigure (**a**) presents a reconstruction of the undersampled stethoscope measurements with an undersampling factor *n* of 3. Subfigure (**b**) is a close-up of the above signal, to clearly demonstrate that the signals are out of sync. Subfigure (**c**) is the result of the cross-correlation of the two synchronization block pulse captures. This is used to determine the offset between the starting time of both captures, and then synchronize the two diastolic sound captures. In subfigure (**d**) the result of this synchronization shift is presented, whereas subfigure (**e**) offers a closer look on the synchronized signals.

The four channels are reconstructed using the CoSaMP CS reconstruction algorithm. The reconstruction of the captured stethoscope data is presented in Figure 6. Since the two acquisition devices are separately controlled, the data is unsynchronized. To synchronize the two measurements, the proposed approach is to reconstruct the undersampled synchronization block pulse, and cross-correlate the two captures of the synchronization signal. The calculated timeshift between the two signals is then applied to the signal of interest to achieve synchronization as displayed in Figure 6. Since the cross-correlation demonstrates a single peak with a sufficient difference in amplitude with other peaks in the data, the conclusion can be made that synchronization up to the resolution of a single sample is possible within the defined boundaries using the proposed approach. Since the tolerance on the synchronization is use-case dependent, the simulation presents a wide range of possible settings to provide a full image of the possible solutions for the designer’s synchronization issue.

### 6.1. Synchronization Robustness

To justify the chosen settings, a Monte Carlo simulation was elaborated to demonstrate the accuracy and limitations of the proposed method [21].

**Figure 7:**
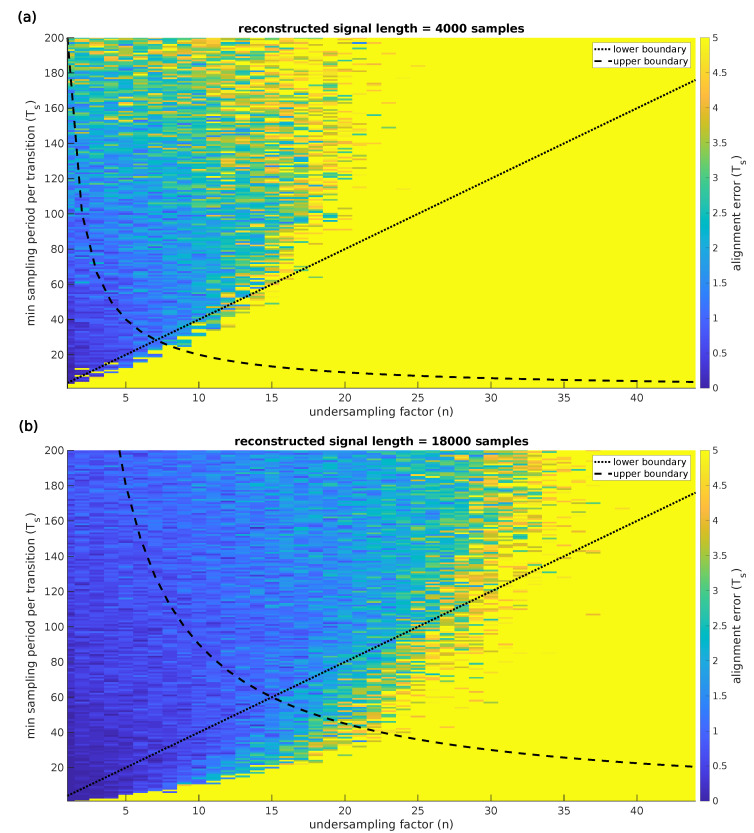
Results of the synchronization alignment error calculation using a Monte Carlo simulation. Subfigure (**a**) presents the maximum alignment error for a signal of 4000 samples, meaning the maximum difference over one-hundred iterations in a calculated timeshift between the synchronization calculation using traditionally sampled signals and the same calculation using undersampled signals. Subfigure (**b**) demonstrates the impact of the signal length on the synchronization accuracy and boundaries by using a signal length of 18,000 samples. Using a larger signal length places the upper boundary higher, leading to a higher achievable maximum undersampling factor with a single sample period synchronization accuracy.

This simulation evaluates the difference in the calculated synchronization timeshift between the traditional Nyquist sampled approach and the CS approach using synchronization signals of 4000 and 18,000 dense samples. The results of this simulation are presented in Figure 7. As a ground truth, the traditional Nyquist sampled data is used. To determine the alignment error, the results obtained using the undersampled signals are compared with the results obtained using the ground truth signal. The simulation runs for one-hundred iterations with a new pseudo-random synchronization signal for every iteration. This synchronization signal is shifted by one-hundred samples to simulate an unsynchronized system with two acquisition devices. The signals are then undersampled with an undersampling factor ranging from one to forty-four. The undersampled signals are then reconstructed and synchronized using the proposed approach. The top panel of Figure 7 presents the maximum alignment error for a signal of 4000 samples, meaning the maximum difference over one-hundred iterations in calculated timeshift between the synchronization calculation using traditionally sampled signals and the same calculation using undersampled signals. The lower boundary for the minimum amount of sample periods per transition is set as four times the undersampling factor. The upper boundary ensures that at least ten transitions are captured, and therefore depends on the amount of acquired signal samples. The lower panel of Figure 7 demonstrates the impact of the signal length on the synchronization accuracy and boundaries by using a signal length of 18,000 samples. Using a larger signal length places the upper boundary higher, leading to a higher achievable maximum undersampling factor with a single sample period synchronization accuracy. This figure clearly demonstrates that the previously mentioned upper and lower boundaries provide appropriate limits to ensure valid synchronization with an accuracy up to one sample period. The maximum alignment error proves to be a good measure for the synchronization accuracy and can therefore be used by the system designer to make a well-founded parameter selection in the model. The comparison with the ground truth signal demonstrates that the proposed approach enables synchronization up to a single clock period when the parameters are selected within the set boundaries.

## 7. Future Work

### 7.1. Model-Based Simulation and Validation

In the research of Bialy et al. [27] the importance of automatic code generation is emphasized as being crucial to the cost-effectiveness of development. The use of model-based code generation accelerates the process whilst decreasing the chance of errors in comparison with manual coding from requirements or models. The use of automatic code generation enables a variety of applications including Software in the Loop, Processor in the Loop, Hardware in the Loop and rapid prototyping [27]. This approach has the advantage of being able to proceed faster between development stages, whilst having a model as a solid foundation for testing and simulation purposes. When implementing simulations and testing in the different development stages, the system behavior can be verified and validated with respect to the key acquisition parameters, such as sampling rate, effective number of bits (ENOB), compression ratio, etc. [28,29]. In our use-case, this model requires a specific choice for an ADC and/or DAC component, as well as other external components, which have to be modeled. This model can be incorporated into a library like the one created by Mertens et al. [30]. This is a Simulink library of embedded platform components for simulation of real-time embedded systems. Expansion of this library for a wide choice of components with their respective characteristics provides the users with a large range of trade-offs to consider, which can be validated and evaluated in the subsequent simulation.

### 7.2. Generation of Glue Electronics and PCB Layout

Continuing on the statement that a system engineer should be able to use this framework, an abstraction needs to be made between the user and the hardware matching the user’s needs. Since there is a great variety in ADC and DAC chips and their respective wiring and pin layout, a subset of commonly used chips has to be modeled and integrated in a library framework. The same will be done for the power-supply circuit and interfacing circuitry such as the sampling and reconstruction filters. This selection of components will be combined and modeled, thus allowing a simulation of the system characteristics depending on the chosen combination. For this selection of components, a list of design constraints can be generated, such as trace length matching. These design constraints can then be combined with user requirements to enable the auto-generation of a functional PCB. This process combined with the use of an extensive library of pre-routed system blocks should allow for a smooth development process from model to PCB.

## 8. Conclusions

This paper presents a good improvement towards the usability of CS on embedded platforms. This is done by means of a model-based code generator and a proposed strategy for CS synchronization. By doing so, a big part of the development complexity which typically lies in the embedded implementation of such a complex sampling scheme is removed. The modularity of the generator framework explicitly promotes reusability between different developments, which will only improve when the framework becomes integrated in more projects since the model components are interchangeable without further issues. This allows the development of an extensive plug-and-play selection of acquisition devices and platforms on which to implement these devices. The results presented in Section 5 demonstrate that the framework generates reliable firmware respecting the defined requirements with a significant gain in development time, without necessitating the deep knowledge typically required when manually programming such firmware.

## Data Availability

The resources developed in this study can be found here: https://cosysgit.uantwerpen.be/cosys-opensource/hesy-daq-generator (accessed on 23 August 2022).

## Abbreviations

The following abbreviations are used in this manuscript:

PCB	Printed circuit board
DAQ	Data acquisition system
FPGA	Field Programmable Gate Array
CS	Compressive Sensing
RISC	Reduced Instruction Set Computer
OS	Operating System
ADC	Analog-to-digital Converter
DAC	Digital-to-analog Converter
RIP	Restricted Isometry Property
ENOB	Effective Number of Bits
NOP	No Operation
